# Life cycle stages of *Hepatozoon ingwe* (Apicomplexa: Adeleorina: Hepatozoidae) in an *Ixodes* sp. tick vector (Arthropoda: Ixodida: Ixodidae) and an African leopard *Panthera pardus pardus*^☆^

**DOI:** 10.1016/j.ijppaw.2025.101049

**Published:** 2025-02-20

**Authors:** Michelle van As, Edward C. Netherlands, Johann van As, Courtney A. Cook, Nico J. Smit

**Affiliations:** a Department Zoology and Entomology, University of the Free State, Qwaqwa Campus, Phuthaditjhaba, 9866, South Africa; b Water Research Group, Unit for Environmental Sciences and Management, North-West University, Potchefstroom Campus, Potchefstroom, 2520, South Africa; c Department of Zoology and Entomology, University of the Free State, Bloemfontein Campus, 9300, South Africa

**Keywords:** *Hepatozoon*, African leopard, Tick vector, Developmental stages

## Abstract

Intracellular apicomplexan haemoparasites from the genus *Hepatozoon* Miller 1908 have been described from a wide range of vertebrate hosts, including wild carnivores in Africa. Reports from the African leopard, *Panthera pardus pardus* (Linnaeus 1758) are scarce and generally non-specific, and description on the mode of transmission and life cycle stages in infected vectors remains relatively rare. The aim of this study was to explore the role of ticks as potential vectors of a species of *Hepatozoon* infecting African leopards in South Africa. Peripheral blood samples and engorged ticks were collected from five wild leopards (three females and two males) while under sedation. Giemsa stained smears of peripheral blood were screened for *Hepatozoon* gamont stages, both extra- and intraleukocytic. Engorged ticks from infected leopards were subsequently kept alive in a fasting state for seven days before being dissected and smeared on clean microscope slides, stained with Giemsa solution, and screened for various possible developmental stages. Sporogonic stages, including microgametes, immature and mature oocysts and infective sporozoites, were observed in a tick (*Ixodes* sp.) collected from a male leopard infected with gamont stages of *Hepatozoon ingwe* Van As, Netherlands and Smit 2020. Developmental stages were photographed, differentiated and measured with ImageJ software. One tick-smear microscope slide was scraped and used for genetic confirmation of the identity of this haemogregarine. This is the first report on the characteristics of different developmental stages of a feline species of *Hepatozoon* in both its potential tick vector and African leopard host.

## Highlights

• This research details the complex life cycle and transmission dynamics of *Hepatozoon ingwe* in leopards and tick vectors.
• Findings confirm an *Ixodes* sp. as vector for *Hepatozoon ingwe*, advancing knowledge of haemoparasite infections in carnivores.
• Detailed morphological characterization of *Hepatozoon ingwe* developmental stages within *Ixodes* tick vectors is presented.
• This research examines potential species-specificity of *Ixodes* ticks as vectors of felid *Hepatozoon* species.
• Likely transmission of *Hepatozoon ingwe* to leopards include ingestion of infected vectors during allo-grooming or predation.

## Graphical abstract

**Figure:**
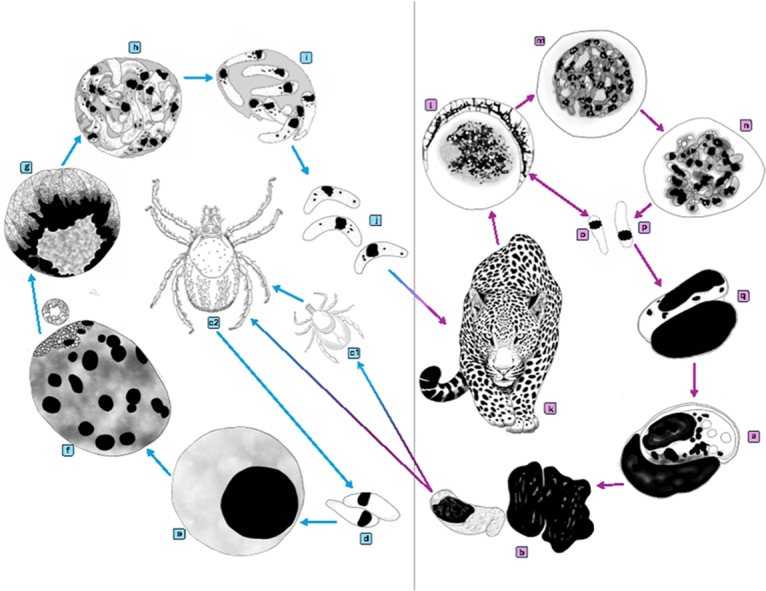
Life cycle stages of *Hepatozoon ingwe* in leopard and tick hosts.

## 1 Introduction

Intracellular haemogregarines from the genus *Hepatozoon* (Phylum Apicomplexa, Suborder Adeleorina, Family Hepatozoidae) infect a wide array of vertebrate hosts (see Smith, 1996). Hepatozoonosis has been reported from several wild mammalian carnivore species (Order Carnivora) in Africa, such as spotted hyaenas *Crocuta crocuta* (Wenyon, 1926; Brocklesby and Vidler, 1963, 1965; Krampitz et al., 1968; Basson et al., 1971; McCully et al., 1975; East et al., 2008), cheetahs *Acinonyx jubatus* (Basson et al., 1971; McCully et al., 1975) and lions *Panthera leo* (Brocklesby and Vidler, 1963, 1965; Krampitz et al., 1968; Basson et al., 1971; McCully et al., 1975). Infections have also been reported from ungulates such as nyala *Tragelaphus angasii* (Basson et al., 1971) and impala *Aepyceros melampus* (Basson et al., 1967), which all form part of the potential prey basis of African carnivores.

Feline hepatozoonosis was first reported from a domestic cat in India during the early 1900s (Patton, 1908), described as an intraleukocytic parasite *Leucocytozoon felis domestici* Patton 1908. Thereafter it was reassigned to the genus *Hepatozoon* (Wenyon 1926) and accepted as *Hepatozoon felis* ( Patton 1908). Until a decade ago, it was thought that wild and domestic felines are generally infected with *H. felis* (Baneth et al., 2013), but a more intricate species complex has been shown through descriptions of *Hepatozoon silvestris* Hodžić, Alić, Prašović, Otranto, Baneth and Duscher 2017 in a European wild cat *Felis silvestris silvestris* (Hodžić et al., 2017) and co-infections of *Hepatozoon luiperdjie* Van As, Netherlands and Smit 2020 and *Hepatozoon ingwe* Van As, Netherlands and Smit 2020 in African leopards *Panthera pardus pardus* in South Africa (Van As et al., 2020). Van As et al. (2020) found that *H. ingwe* infected the leopard's lymphocytes, usually compacted lymphocyte nuclei towards one side and completely usurped lymphocyte cytoplasm. *Hepatozoon ingwe* is also closely related to *H. silvestris* (see Van As et al., 2020).

A completely and well-documented life cycle, which includes morphological data from the haematophagous host (or definitive host), is only available for a few species of *Hepatozoon* (Smith, 1996; Baneth et al., 1998, 2007; Van As et al., 2015). Species of *Hepatozoon* typically follow a two host life cycle, involving sporogonic development and oocyst formation in the definitive haematophagous invertebrate host, and merogony and gametogony in the intermediate vertebrate host (Siddall, 1995; Smith, 1996; Telford, 2009). Definitive hosts of species of *Hepatozoon* include mites, ticks, tsetse flies, sand flies, mosquitoes, fleas, leeches, lice and reduviid bugs (Smith, 1996). Transmission to an intermediate vertebrate host is generally accomplished through ingestion of a mature, oocyst-containing, definitive arthropod host (McCully et al., 1975; Davies and Johnston, 2000; Baneth et al., 2007; Van As et al., 2015), but it has also been proven that infection through inoculation is possible by predation on other infected intermediate vertebrate hosts (Landau et al., 1972; Smith et al., 1994; Smith, 1996; Ewing et al., 2002; Baneth, 2011). Grooming behaviour may also facilitate haemogregarine infections (Ewing et al., 2002; East et al., 2008) and transplacental transmission has been reported in *Hepatozoon canis* infections in mammals (Murata et al., 1993). Baneth et al. (2013) also suggested the possibility of transplacental transmission of *H. felis* in domestic cats.

It has been reported that *Hepatozoon griseisciuri* and *Hepatozoon americanum* sporocysts rupture to release sporozoites upon contact with bile (Redington and Jachowski, 1971; Mathew et al., 1999). It is unknown how species of *Hepatozoon* sporozoites migrate through a carnivore body following ingestion of an infected haematophagous vector. Baneth et al. (2007) proposed some interesting theories in this regard, all of which remain to be confirmed. Infective sporozoites are released in the intermediate host's gut and penetrate the intestinal wall from where the host's bloodstream carries them to become meronts in the bone marrow, spleen, lungs, liver, lymph nodes and myocardium (McCully et al., 1975; Craig et al., 1978; O'Dwyer, 2011). Giannelli et al. (2017) found that sporozoites react to exposure to bile by starting to move in a gliding and flexing manner. It has been shown that the schizogonic cycle of *H. canis* starts in the domestic dog's intestinal wall (Forlano et al., 2005). The *Hepatozoon* gamont stage infects erythrocytes or leukocytes of the vertebrate host, depending on the identity of the host. Reptile erythrocytes and mammalian and avian leukocytes are typically infected by gamont stages (Smith, 1996; Telford, 2009; Baneth, 2011). Small merogonic cysts in vertebrate host tissues, containing various parasitic stages, have been reported for several *Hepatozoon* species (Smith, 1996) and these cysts are considered to be mainly associated with infections incurred by predation (Smith et al., 1994; Vincent-Johnson et al., 1997; Paperna et al., 2002; Baneth et al., 2003, 2007). It also seems that the locality of these cysts may be species-specific to its infecting haemogregarine (Baneth, 2011). In mammal hosts, merozoites are released from meronts and invade leukocytes that enter the peripheral bloodstream, where development into gamonts takes place (McCully et al., 1975; O'Dwyer, 2011). Haematophagous ticks ingest *Hepatozoon*-containing leukocytes during a blood meal and the gamonts subsequently exit the leukocytes within the gut of the tick. Here, gamonts group into pairs and differentiate into micro- and macrogametes, which fuse to form a zygote. Zygotes then undergo sporogony to develop into mature oocysts, containing many sporocysts with infective sporozoites in the haemocoel of the tick (Baneth et al., 2001, 2003, 2007). Telford (2009) suggested that the number and morphometrics of intrasporocystic sporozoites could be characteristic of a species of *Hepatozoon*.

The vectors of many species of *Hepatozoon* are still unknown and need detailed research (Smith, 1996; Forlano et al., 2005; Baneth, 2011). Currently, to the authors' best knowledge, all known life cycles of species of *Hepatozoon* infecting mammalian carnivores have an ixodid tick definitive host (Table 1). The development of *H. canis* was first described in *Rhipicephalus sanguineus* by Christophers (1907) and Wenyon (1911). Current mammalian carnivore-associated vector and life cycle stages have only been reported for *H. canis* (Baneth et al., 2007; Rubini et al., 2009; de Miranda et al., 2011; O'Dwyer, 2011; Giannelli et al., 2013a, 2013b, 2017), *H. americanum* (Mathew et al., 1998; Ewing et al., 2002), *Hepatozoon ursi* and *Hepatozoon martis* from Japanese martens (Hodžić et al., 2018). Various tick species such as *Haemaphysalis longicornis* Neumann 1901*, Haemaphysalis hystricis* Supino 1897*, Haemaphysalis megaspinosa* Saito 1969*, Haemaphysalis campanulata* Warburton 1908*, Amblyomma testudinarium* Koch 1844 and *Ixodes tanuki* Saito 1964 have been reported to contain *H. felis* DNA (Tateno et al., 2015). The DNA of *H. felis* was also detected in *R. sanguineus* ticks collected from humans in Turkey (Aktas, 2014), domestic cats in Portugal (Maia et al., 2014) and captive lions in Thailand (Bhusri et al., 2017).

**Table 1:** Morphometrics of mammalian carnivore *Hepatozoon* sp. developmental stages in their haematophagous definitive hosts. NS: not stated; ☐: not relevant.

Vector	*Hepatozoon* species	Measurements (L±SD × W±SD (Min - Max) or diameter (Min - Max or range (Min - Max)) um	Number measured/counted	Next developmental stage/within previous stage	Number of intracellular developmental stage	Associated intermediate host	Reference
**Immature oocysts**							

*Rhipicephalus sanguineus*	*Hepatozoon canis*	188.3 ± 34.7 ×171.1 ± 40.0	49	☐	☐	*Canis familiaris*	Giannelli et al. (2013b)
*Rhipicephalus sanguineus*	*Hepatozoon canis*	78.8 ± 12.4 × 78.8 ± 12.4	3	☐	☐	*Canis familiaris*	Baneth et al. (2007)
*Rhipicephalus turanicus*	*Hepatozoon canis*	201 ± 72.8 × 138.8 ± 48.6	NS	☐	☐	*Canis familiaris*	Giannelli et al. (2017)

**Maturing oocysts**							

*Amblyomma maculatum*	*Hepatozoon americanum*	269 (188–323)	40	☐	☐	*Canis familiaris*	Ewing et al. (2002)
*Amblyomma maculatum*	*Hepatozoon americanum*	☐	50	Sporocysts within maturing oocyst	311 (91–458) (n = 50 oocysts)	*Canis familiaris*	Ewing et al. (2002)
*Rhipicephalus sanguineus*	*Hepatozoon canis*	252.6 ± 68.4 × 247.3 ± 76	2	☐	☐	*Canis familiaris*	Baneth et al. (2007)

**Mature/sporulated oocysts**							

*Amblyomma maculatum*	*Hepatozoon americanum*	339 (159–438)	50	☐	☐	*Canis familiaris*	Ewing et al. (2002)
*Amblyomma ovale*	*Hepatozoon canis*	244.34 × 255.46	4	☐	☐	*Canis familiaris*	Rubini et al. (2009)
*Rhipicephalus microplus*	*Hepatozoon canis*	251.3 ± 13.7 × 173.9 ± 5.34	8	☐	☐	*Canis familiaris*	de Miranda et al. (2011)
*Rhipicephalus sanguineus*	*Hepatozoon canis*	237.1 ± 27.1 × 226.9 ± 24.0	2	☐	☐	*Canis familiaris*	Giannelli et al. (2013b)
*Rhipicephalus sanguineus*	*Hepatozoon canis*	309.8 ± 31.8 × 255.8 ± 48.0	5	☐	☐	*Canis familiaris*	Baneth et al. (2007)
*Haemaphysalis flava*	*Hepatozoon ursi*	297.5 (263.2–331.8) × 232.85 (231.70–234.0)	2	☐	☐	*Ursus thibethanus japonicus*	Kubo et al. (2008)
*Rhipicephalus turanicus*	*Hepatozoon canis*	259.9 ± 36.1 × 246.1 ± 33.9	NS	☐	☐	*Canis familiaris*	Giannelli et al. (2017)
*Amblyomma maculatum*	*Hepatozoon americanum*	390.0 ± 59.9 × 35.6 ± 58.9 (310–480 × 260–460)	24	☐	☐	*Canis familiaris*	Vincent-Johnson et al. (1997)
*Rhipicephalus sanguineus*	*Hepatozoon canis*	214.8 ± 45.5 × 192.9 ± 36.5 (160–325 × 138–258)	15	☐	☐	*Canis familiaris*	Vincent-Johnson et al. (1997)
*Haemaphysalis longicornis*	*Hepatozoon canis*	300 × 150	2	☐	☐	*Canis familiaris*	Murata et al. (1995)
*Amblyomma ovale*	*Hepatozoon canis*	214 × 209	2	☐	☐	*Canis familiaris*	Forlano et al. (2005)
*Amblyomma maculatum*	*Hepatozoon americanum*	294 ± 43 (175–495)	100	☐	☐	*Canis familiaris*	Mathew et al. (1999)
*Amblyomma maculatum*	*Hepatozoon americanum*	225 ± 37 (145–405)	100	☐	☐	*Canis familiaris*	Mathew et al. (1999)
*Amblyomma maculatum*	*Hepatozoon americanum*	300–1000	20	☐	☐	*Canis familiaris*	Mathew et al. (1998)
*Ixodes* sp.	*Hepatozooningwe*	190.88 ± 16.35 (179.52–209.62) × 157.74 ± 18.78 (136.39–171.76)	3	☐	☐	*Panthera pardus pardus*	Current study

**Intraoocysctic sporocysts**							

*Rhipicephalus sanguineus*	*Hepatozoon canis*	25.5 ± 2.9 × 20.2 ± 5.8	NS	☐	☐	*Canis familiaris*	Giannelli et al. (2013b)
*Rhipicephalus sanguineus*	*Hepatozoon canis*	32.3 ± 0 x 13.95 ± 1.5	2	☐	☐	*Canis familiaris*	Baneth et al. (2007)
*Haemaphysalis flava*	*Hepatozoon ursi*	31.2 ± 2.5 ×27.0 ± 2.9 (28.0–34.6 × 23.7–32.0)	5	☐	☐	*Ursus thibethanus japonicus*	Kubo et al. (2008)
*Haemaphysalis flava*	*Hepatozoon ursi*	☐	2	Sporocysts within mature oocysts	45 (40–50) (n = 2 oocysts)	*Ursus thibethanus japonicus*	Kubo et al. (2008)
*Rhipicephalus turanicus*	*Hepatozoon canis*	32.1 ± 4.7 × 20.2 ± 2	NS	☐	☐	*Canis familiaris*	Giannelli et al. (2017)
*Haemaphysalis longicornis*	*Hepatozoon canis*	30 × 30	NS	☐	☐	*Canis familiaris*	Murata et al. (1995)
*Haemaphysalis longicornis*	*Hepatozoon canis*	☐	2	Sporocysts within mature oocysts	50–70 (n = 2 oocysts)	*Canis familiaris*	Murata et al. (1995)
*Amblyomma ovale*	*Hepatozoon canis*	3.5 × 21.5	NS	☐	☐	*Canis familiaris*	Forlano et al. (2005)
*Amblyomma ovale*	*Hepatozoon canis*	☐	2	Sporocysts within mature oocysts	240 (n = 2 oocysts)	*Canis familiaris*	Forlano et al. (2005)
*Amblyomma maculatum*	*Hepatozoon americanum*	27.8 ± 4.8 (18–39)	100	☐	☐	*Canis familiaris*	Mathew et al. (1999)
*Amblyomma maculatum*	*Hepatozoon americanum*	☐	20	Sporocysts within mature oocysts	656 ± 176 (260–1040) (n = 20 oocysts)	*Canis familiaris*	Mathew et al. (1999)

**Sporozoites within intraoocystic sporocysts**							

*Haemaphysalis flava*	*Hepatozoon ursi*	12.2 ± 1.4 ×3.5 ± 0.5 (10.0–14.0 × 2.9–4.2)	4	☐	☐	*Ursus thibethanus japonicus*	Kubo et al. (2008)
*Haemaphysalis flava*	*Hepatozoon ursi*	☐	4	Sporozoites within intraoocystic sporocysts	8–16 (n = 4 intraoocystic sporocysts)	*Ursus thibethanus japonicus*	Kubo et al. (2008)
*Amblyomma ovale*	*Hepatozoon canis*	☐	NS	Sporozoites within intraoocystic sporocysts	20 (n = NS)	*Canis familiaris*	Forlano et al. (2005)
*Amblyomma maculatum*	*Hepatozoon americanum*	15 ± 1.2 × 5	NS	☐	☐	*Canis familiaris*	Mathew et al. (1999)
*Amblyomma maculatum*	*Hepatozoon americanum*	☐	15	Sporozoites within intraoocystic sporocysts	10–26 (n = 15 intraoocystic sporocysts)	*Canis familiaris*	Mathew et al. (1999)

**Free Sporocysts**							

*Amblyomma maculatum*	*Hepatozoon americanum*	25 (20–31)	50	☐	☐	*Canis familiaris*	Ewing et al. (2002)
*Amblyomma maculatum*	*Hepatozoon americanum*	☐	28	Sporozoites within free sporocysts	18 (12–26) (n = 28 sporocysts)	*Canis familiaris*	Ewing et al. (2002)
*Rhipicephalus sanguineus*	*Hepatozoon canis*	37.0 ± 4.2 ×21.6 ± 1.5	NS	☐	☐	*Canis familiaris*	Giannelli et al. (2013b)
*Amblyomma maculatum*	*Hepatozoon americanum*	26.1 ± 2.0 × 24.8 ± 1.8 (20–30 × 20–29)	58	☐	☐	*Canis familiaris*	Vincent-Johnson et al. (1997)
*Rhipicephalus sanguineus*	*Hepatozoon canis*	35.6 ± 3.7 × 25.7 ± 2.8 (29–41 ×17–30)	31	☐	☐	*Canis familiaris*	Vincent-Johnson et al. (1997)
*Ixodes* sp.	*Hepatozoon ingwe*	30.3 ± 2.0 (26.5–34.2)× 29.8 ± 2.3 (24–32.8)	20	☐	☐	*Panthera pardus pardus*	This study
*Ixodes* sp.	*Hepatozooningwe*	☐	14	Sporozoites within free sporocysts	30 ± 6 (21–41) (n = 14 sporocysts)	*Panthera pardus pardus*	This study
*Haemaphysalis longicornis*	*Hepatozoon canis*	☐	NS	Sporozoites within free sporocysts	10–16 (n = NS)	*Canis familiaris*	Murata et al. (1995)
*Amblyomma maculatum*	*Hepatozoon americanum*	20–30	20	☐	☐	*Canis familiaris*	Mathew et al. (1998)
*Amblyomma maculatum*	*Hepatozoon americanum*	☐	20	Sporozoites within free sporocysts	8–16 (n = 20 sporocysts)	*Canis familiaris*	Mathew et al. (1998)

**Intrasporocystic sporozoites in free sporocysts**							

*Ixodes* sp.	*Hepatozooningwe*	12.86 ± 4.22 (6.56–19.92) × 2.00 ± 0.60 (1.17–2.81)	9	☐	☐	*Panthera pardus pardus*	This study
*Haemaphysalis longicornis*	*Hepatozoon canis*	10 x 3	NS	☐	☐	*Canis familiaris*	Murata et al. (1995)

**Intrasporocystic sporozoite nuclei**							

*Ixodes* sp.	*Hepatozooningwe*	1.72 ± 0.56 (0.93–3.4) × 1.65 ± 0.46 (2.00–2.60)	23	☐	☐	*Panthera pardus pardus*	This study

**Free swimming mature sporozoites**							

*Rhipicephalus turanicus*	*Hepatozoon canis*	15.5 ± 4.1 × 3 ± 0.6	NS	☐	☐	*Canis familiaris*	Giannelli et al. (2017)
*Amblyomma maculatum*	*Hepatozoon americanum*	13–17 × 4–7	20	☐	☐	*Canis familiaris*	Mathew et al. (1998)
*Ixodes* sp.	*Hepatozooningwe*	17.11 ± 1.22 (14.89–20.11) × 3.09 ± 0.47 (2.07–4.19)	22	☐	☐	*Panthera pardus pardus*	This study

**Nuclei of free swimming mature sporozoites**							

*Ixodes* sp.	*Hepatozooningwe*	2.92 ± 0.44 (2.16–3.67) × 2.81 ± 0.57 (1.91–3.79)	22	☐	☐	*Panthera pardus pardus*	This study

Most studies that include the life cycle stages of *Hepatozoon* species in engorged ticks collected from infected carnivores mainly focused on the presence of oocysts in the tick, often without exploring further developmental stages. This is most probably due to the widely accepted idea that oocysts serve as the infective stage for the intermediate hosts or vectors (Murata et al., 1995; Mathew et al., 1999; Ewing et al., 2002; Forlano et al., 2005; Baneth et al., 2007; Rubini et al., 2009; Baneth, 2011; de Miranda et al., 2011; Demoner et al., 2013; Giannelli et al., 2013a). However, a few studies, such as Giannelli et al. (2013b), report further developmental stages of *H. canis* in *R. sanguineus* ticks. To date only two studies have described the presence of free-swimming sporozoites in ticks; Forlano et al. (2005) who reported free sporozoites of *H. canis* in *Amblyomma ovale* Koch 1844, and Giannelli et al. (2017) who found free sporozoites of *H. canis* in *Rhipicephalus turanicus* Pomerantsev 1936. Furthermore, various studies only report on the presence of oocysts and not their morphometrics. During a study on hepatozoonosis in spotted hyaenas, lions, jackals, cheetahs and one leopard in South Africa's Kruger National Park, McCully et al. (1975) reported sporogenous development of a *Hepatozoon* sp. in the haemolymph smears of *Rhipicephalus simus* Koch, 1844 females from a spotted hyaena, with no development in *R. sanguineus* or *Haemaphysalis leachi* (Audouin, 1826) ticks collected from the same hyaena. However, McCully et al. (1975) did find that *R. sanguineus* are successful vectors for a species of *Hepatozoon* infecting black-backed jackals, but no morphometrics was reported in their paper. Other studies reported the detection of carnivore-related hepatozoans in ixodid ticks without providing morphometric data. These include Demoner et al. (2013), who reported genetic detection and oocysts of *H. canis*; Bhusri et al. (2017), who only reported genetic detection of *H. felis*; Hodžić et al. (2018), who only reported genetic detection of *H. martis*; and Uiterwijk et al. (2023), who reported the presence of several *Hepatozoon* spp. from different tick species by means of molecular analyses.

The clinical properties of feline hepatozoonosis are currently still poorly understood (Kegler et al., 2018) and the potential vectors for feline hepatozoonosis remain relatively unknown (Baneth et al., 2013; Lloret et al., 2015). Little is currently known about the host-specificity of species of *Hepatozoon* in African carnivores, even though it has been suggested by Redington and Jachowski (1971) that more studies should focus on the developmental stages in associated invertebrate hosts. Following the reasoning of Baneth et al. (2013), it is expected that this haemogregarine is most likely transmitted by haematophagous arthropods, just like other species of the genus *Hepatozoon*. Duplan et al. (2018) obtained and reported the 18S rDNA sequence data of *H. silvestris* from an *Ixodes ricinus* removed from a domestic cat and concluded that this species may serve as a potential vector for this haemogregarine. Our study investigated whether ticks collected from an African leopard infected with *H. ingwe* serve as potential vectors for this species of *Hepatozoon*. This paper provides the first detailed morphological descriptions of *H. ingwe* life cycle stages in its wild-collected haematophagous definitive host, an *Ixodes* sp. tick (Arthropoda: Ixodida: Ixodidae). This is the first report of its kind for a leopard-associated *Hepatozoon* species.

## 2 Materials and methods

Data collection took place in the Mpumalanga Province of South Africa (25°9′51.31ʺS, 30°26′55.46ʺE) (24°34′43.50ʺS, 31°25′46.48ʺE). Leopards were captured, sedated and sampled by a qualified veterinarian following standard procedures as prescribed and approved by a National Registered Animal Ethics committee (North-West University ethics approval no. NWU-00255-17-A5). Blood collection and peripheral blood smears were as described by van As et al. (2020).

Engorged ticks were collected from a male leopard that was reported to be infected by *H. ingwe* (see Van As et al., 2020). Ixodid ticks can stay attached to their hosts for several days while feeding (Walker et al., 2003) and early oocysts of *H. canis* have been observed in ticks within one to four days after removal of an engorged tick from canid hosts (Baneth et al., 2007; Rubini et al., 2009; O'Dwyer, 2011). Considering this, engorged ticks were dissected only after seven days of being kept in a fasting state in order to allow observation of further developmental stages. Ticks were ventrally incised from posterior to anterior to expose gut contents and haemolymph. Gut contents containing haemolymph were then smeared on clean microscope slides, fixed in absolute methanol for 1 min, air-dried and stained with 10% Giemsa (Sigma-Aldrich®) solution for 20 min according to Van As et al. (2015). Peripheral blood and tick smears were screened with the aid of a Nikon Eclipse E800 compound microscope (Nikon®, Amsterdam, The Netherlands) under a 40x for possible *Hepatozoon* life stages. Identified stages were photographed with an attached Nikon DS-Fi1 digital camera and accompanying software. Identified and clearly visible developmental life cycle stages (and their subsequent characteristics) were digitally measured with the ImageJ version 1.47 software program (Wayne Rasband National Industries of Health, USA) (Rasband, 2014) (http://imagej.nih.gov/ij).

### 2.1 Molecular analysis

Genomic DNA extraction was performed on a single methanol-fixed, Giemsa-stained tick smear microscopically confirmed to contain *Hepatozoon* developmental stages. The extraction followed the scraping procedure outlined by Cook et al. (2014), and DNA was isolated using the standard protocol for nucleated blood cells detailed in the Quick-DNA Microprep Plus Kit (Zymo Research, USA). Polymerase Chain Reaction (PCR), sequencing, and sequence assembly were carried out as per the methodology described by Netherlands et al. (2021) for the HepF300 and HepR900 primer sets. The validation of sequence identity was executed by comparing sequences to existing records in GenBank^TM^, utilising the Basic Local Alignment Search Tool (BLAST) (Altschul et al., 1990). Nine 18S rDNA gene sequences from closely related *Hepatozoon* species, including *Hepatozoon ingwe* (MN793000 - MN793001), were retrieved from GenBank and aligned with the sequence produced in this study using the MUSCLE alignment tool (Edgar, 2004) within Geneious R11 (http://www.geneious.com, Kearse et al., 2012). Uncorrected pairwise distances (p–distance) were calculated using PAUP version 4.0a (Swofford, 2002).

### 2.2 Morphological identification of *Hepatozoon* life cycle stages

A compilation of several literature sources was used in the identification of possible life cycle stages of *Hepatozoon* species (Table 2). Our study followed the suggestion by Giannelli et al. (2013a) that the presence of *Hepatozoon* sp. oocysts in a tick vector collected from a known infected carnivore with the same *Hepatozoon* sp. infection is indicative that the tick may be a potential vector for that *Hepatozoon* species. Ticks were identified using Walker et al. (2003) and tick specialists at The Agricultural Research Council-Onderstepoort Veterinary Research Campus (ARC-OVR), South Africa confirmed identification.

**Table 2:** Morphological characteristics of various *Hepatozoon* life cycle stages (mainly compiled from Smith (1996); Telford (2009)).

Life cycle stage	Morphological description	Additional References
Immature oocyst	Near-spherical in shape with a dense central matrix, surrounded by lighter staining basophilic cytoplasm and visibly evident outer membrane. No sporocysts formed.	Baneth et al. (2007) Giannelli et al. (2013b, 2017)
Maturing oocysts	Early sporocyst formation visible as nonhomogenic cytoplasm. Thick visible membrane.	Ewing et al. (2002) Baneth et al. (2007) Van As et al. (2015)
Mature oocysts	Appear as a thin structure encompassing sporocysts. Sporulated with defined sporocysts containing developing sporozoites. Thin membrane. Considered the infective stage to intermediate vertebrate host.	Vincent-Johnson et al. (1997) Ewing et al. (2002) Baneth et al. (2007)de Miranda et al. (2011) Giannelli et al. (2013b)
Sporocysts	Round to ellipsoidal within maturing or mature oocysts; sometimes free in tick haemocoel. Membrane wall could be thick or thin. Internal cytoplasm foamy in young sporocysts, endopolygenous development into intrasporocystic sporozoites. Mostly intraoocystic, could be free in haemolymph.	Murata et al. (1995) Mathew et al. (1999) Baneth et al. (2007) Kubo et al. (2008) Giannelli et al. (2013a, 2017)
Sporozoites	Elongated, narrow, curved, slightly banana shaped with foamy or lightly stained cytoplasm and dense nuclei. Thickly packed within sporocyst with no particular arrangement. Thin membrane. Mostly intrasporocystic, could be free in haemolymph.	Murata et al. (1995) Mathew et al. (1998, 1999) Forlano et al. (2005) Kubo et al. (2008) Giannelli et al. (2017)

## 3 Results

**Fig. 1:**
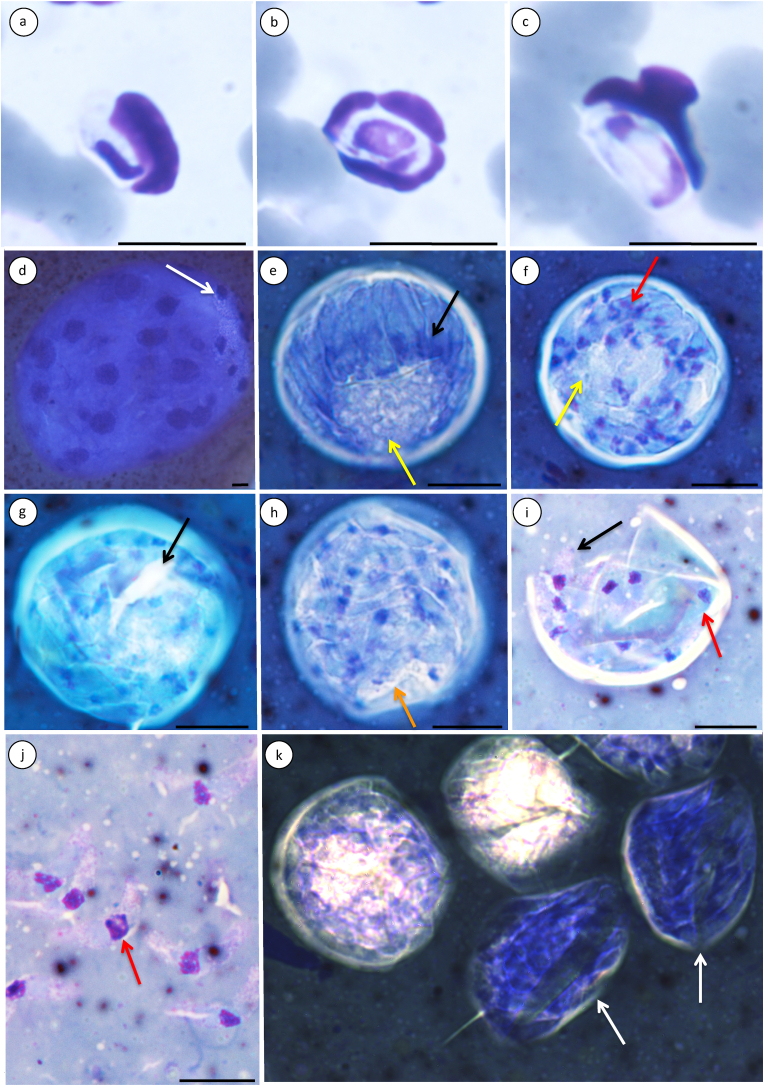
Developmental stages of *Hepatozoon ingwe* in the peripheral blood of an African leopard *Panthera pardus pardus* and the gut contents of the *Ixodes* tick. **a–c:** Gamont stages of *Hepatozoon ingwe* in peripheral bloodsmears of the infected leopard; **a:** Immature gamont. **b:** Mature gamont. **c:** Double-folded mature gamont. **d–k:** Sporogonic stages in an *Ixodes* tick; **d:** Mature, multinucleate sporulated oocyst with 19 nuclei in the tick's haemocoel. See intracellular developing sporocysts apexally aggregating as indicated by white arrow; **e–g:** Maturing sporocysts at various stages of development. Note how the contained sporozoites mature from e to g; **e:** Free-lying sporocyst containing early separating, immature sporozoites; Sporocyst encapsulated in distinct white membrane. Early invagination of sporozoites, separated by thin, dark indigo membranes. Immature sporozoites separate at apexal area and crown basal mass, seen as light cobalt blue area with a foamy appearance (yellow arrow). Immature sporozoite cytoplasm uniform azure/sky blue. Apexal aggregation of chromatin, seen as dark indigo spherical shapes near sporozoite apexes (black arrow). **f:** Immature sporozoites. Cytoplasm is turquoise and grainy, containing dense dark blue chromatin, some with purple and magenta granules (red arrow). Small section of light blue basal mass visible (yellow arrow). Sporozoites more evenly distributed throughout sporocyst with relaxing crown formation. Sporocyst membrane distinctly white. **g:** Sporocyst with membrane lysis, containing developing sporozoites with the same staining properties as in 1 f. Membrane turquoise colored and looser around the sporocyst. Sporozoites evenly distributed throughout sporocyst and cytoplasm of some sporozoites stain grainy turquoise. Spongy basal mass completely absorbed, leaving a white area (black arrow). **h:** Mature sporocyst with lysed membrane. Sporozoites distributed throughout whole sporocyst. Sporozoite cytoplasm grainy light blue, sometimes very foamy light blue (orange arrow), with distinct white membranes. Nuclei dense dark blue chromatin with purple granules, rounded, centralized. **i:** Ruptured sporocyst with distinct white membrane, exuding mature sporozoites (black arrow). Sporozoite cytoplasm grainy light pink and purple, no visible membrane, tapered towards extremities. Chromatin not dense, purple with magenta and dark purple granules, situated towards center of sporozoite. Sporozoites still seen through sporocyst membrane (red arrow) has overall bluer hue than exuding sporozoites (black arrow). **j:** Free-swimming mature sporozoites suspended in tick's haemocoel. Staining properties similar to exuding sporozoites in 1 i (black arrow). Membrane not clearly visible. Tapered towards both extremities. Chromatin loosely concentrated as rectangular shape in middle of mature sporozoite with more magenta granules than in 1 i (black arrow). **k:** Multiple sporocysts at various stages of development. Scale bar indicates 10 μm.

All *Hepatozoon ingwe* sporogonic stages were found within a single hard-bodied tick, identified as an unknown *Ixodes* Latreille, 1795 species (Order Ixodida; Family Ixodidae) (Fig. 1) (Table 1). A 616-nucleotide amplicon of the nuclear 18S rRNA gene was obtained from this tick smear, collected from a *Panthera pardus pardus* specimen infected with *H. ingwe* (**MN793001**) (Van As et al., 2020). Comparison with available 18S rDNA sequences on GenBank through BLAST analysis revealed a robust 99.51% identity to *H. ingwe* sequences. Furthermore, the uncorrected pairwise distance (p–distance) of 0.49% confirmed the identification of the isolated species as *H. ingwe* (GenBank: **MN793000** - **MN793001**) (Table 3). Other ticks collected from this leopard included *Haemaphysalis elliptica* (Koch, 1844) (Order Ixodida; Family Ixodidae; Subfamily Haemaphysalinae), *Hs. leachi* (Order Ixodida; Family Ixodidae; Subfamily Haemaphysalinae), *R. simus* (Order Ixodida; Family Ixodidae) and an unknown *Haemaphysalis* Koch 1844 species. No *Hepatozoon* species developmental stages were observed in these ticks.

**Table 3:** Estimates of evolutionary divergence between partial 18S rDNA sequences from *Hepatozoon* species closely related to *Hepatozoon ingwe* (MN793000 - MN793001). Matrix showing uncorrected p-distance per site between sequences. Numbers presented as percentage (%).

	1.	2.	3.	4.	5.	6.	7.	8.	9.	10.
1. ***Hepatozoon ingwe* ex *Ixodes* sp. ex *Panthera pardus pardus***										
2. MN793001 *Hepatozoon ingwe* ex *Panthera pardus pardus*	0.49									
3. MN793000 *Hepatozoon ingwe* ex *Panthera pardus pardus*	0.49	0.00								
4. OM256568 *Hepatozoon* sp. ex *Felis silvestris*	0.49	0.32	0.32							
5. ON075470 *Hepatozoon felis* ex *Panthera leo persica*	0.65	0.49	0.49	0.49						
6. KC138533 *Hepatozoon felis* ex *Felis catus*	0.65	0.49	0.49	0.49	0.00					
7. KC138534 *Hepatozoon felis* ex *Felis catus*	0.65	0.49	0.49	0.49	0.00	0.00				
8. AY620232 *Hepatozoon felis* ex *Felis catus*	0.81	0.65	0.65	0.65	0.16	0.16	0.16			
9. MN793003 *Hepatozoon luiperdjie* ex *Panthera pardus pardus*	1.46	1.30	1.30	1.30	1.46	1.46	1.46	1.62		
10. MN793002 *Hepatozoon luiperdjie* ex *Panthera pardus pardus*	1.46	1.30	1.30	1.30	1.46	1.46	1.46	1.62	0.00	

Peripheral blood smears of this particular leopard had gamont stages of *H. ingwe* at various levels of maturity. The infection in this specific leopard was previously documented both morphologically and molecularly by Van As et al. (2020) (Fig. 1a–c). Given that this leopard was infected with *H. ingwe* (Van As et al., 2020) and that the isolated DNA obtained from the tick smear collected from this leopard demonstrated a 99.51% similarity to *H. ingwe*, it is reasonable to conclude that the observed sporogonic stages in the tick smear correspond to *H. ingwe*.

### 3.1 Description of merogonic and gamont stages in a wild African leopard

Fig. 1a–c.

No merogonic stages were observed, as live leopards were sampled and no necropsied specimens were available for this study (see Van As et al., 2020). Gamont stages in peripheral blood smears shown in Fig. 1 a to c is that of *Hepatozoon ingwe*, at immature (Fig. 1 a) and mature (Fig. 1 b and 1 c) stages. Detailed morphological descriptions of these stages are given in Van As et al. (2020).

### 3.2 Description of sporogonic stages in a wild-collected, engorged *Ixodes* sp. tick

Fig. 1d–k.

Stages found within the haemocoel of an *Ixodes* sp. tick included maturing sporulated oocysts, free sporocysts with growing sporozoites at various stages of development, mature sporocysts with mature sporozoites, ruptured sporocysts with exuding sporozoites and free-swimming sporozoites. No gametogenesis and subsequent fertilization was observed. Development of sporogonic stages, with corresponding micrographs (Fig. 1), are described below.

**Mature, multinucleate sporulated oocysts (**Fig. 1 d**):** Suspended free in the haemocoel. Spherical/oval shape. Mature oocysts measured 190.88 ± 16.35 (179.52–209.62) x 157.74 ± 18.78 (136.39–171.76) *μ*m, area 23302.27 ± 3035.21 (19932.52–25821.45) *μ*m^2^ (n = 4). Contained 23–25 sporocysts each (n = 4). Faint, thin, dark blue membrane. Cytoplasm uniform purplish blue, more foamy at apical area. Differentiating nuclei (average 19 per oocyst) equally distributed in cytoplasm, dense and uniform dark indigo, round to oval shape.

**Remarks (oocysts):** Sporulated oocysts were clearly visible in the tick's haemocoel as free-lying dots on Giemsa-stained microscope slides, similar to sporulated oocysts of *H. americanum* (Mathew et al., 1998, 1999) in *Amblyomma maculatum* Koch, 1844 (Vincent-Johnson et al., 1997), *H. canis* (Murata et al., 1995) and an unknown *Hepatozoon* species (McCully et al., 1975). Maturing *Hepatozoon* oocysts have also been reported in *A. maculatum* (*H. americanum*) (Ewing et al., 2002) and *R. sanguineus* (*H. canis*) (Baneth et al., 2007) and *Rhipicephalus turanicus* (*H. canis*) (Giannelli et al., 2017). Similar to the studies of Kubo et al. (2008) and Murata et al. (1995), no immature or ruptured oocysts releasing sporocysts were observed. The number of intraoocystic sporocysts were closest to that of *H. ursi* in *Haemaphysalis flava* Neumann, 1897 (Kubo et al., 2008) (Table 1) and *H. canis* in *Hs. longicornis* (Murata et al., 1995), but far fewer than that of *H. americanum* in *A. maculatum* (Ewing et al., 2002) and *H. canis* in *A. ovale* (Forlano et al., 2005) (Table 1). Sporulation seems to commence at the apical area of oocysts (white arrow in Fig. 1 d), with remaining oocyst nuclei dispersed throughout the rest of the cytoplasm. Sporulated oocysts were oval-shaped, differing from round sporulated oocysts of *H. canis* in adult *A. ovale* ticks (Forlano et al., 2005; Rubini et al., 2009). Oval-shaped sporulated oocysts have, however, been reported for *H. canis* in a naturally infected *Rhipicephalus microplus* (Canestrini, 1888) tick (de Miranda et al., 2011), and in adult *R. sanguineus* (Giannelli et al., 2013b) and *R. turanicus* (Giannelli et al., 2017). Sporulated oocysts' thin membranes were similar to those of *H. canis* in *R. sanguineus* (Baneth et al., 2007) and *Hs. longicornis* (Murata et al., 1995). Sporulated oocysts were morphometrically most similar to immature oocysts of *H. canis* in *R. sanguineus* (Giannelli et al., 2013b), and smaller in size than that of *H. canis* in *A. ovale* (Forlano et al., 2005; Rubini et al., 2009), *R. microplus* (de Miranda et al., 2011), *R. sanguineus* (Vincent-Johnson et al., 1997; Baneth et al., 2007; Giannelli et al., 2013b), *R. turanicus* (Giannelli et al., 2017), *Hs. longicornis* (Murata et al., 1995), *H. americanum* in *A. maculatum* (Vincent-Johnson et al., 1997; Mathew et al., 1998, 1999; Ewing et al., 2002), and *H. ursi* in *Hs. flava* (Kubo et al., 2008) (Table 1). Oocysts tend to enlarge as they mature (Bashtar et al., 1991; Mathew et al., 1999; Baneth et al., 2007), which is evident in the relatively large variation in the sporulated oocyst size in the present study. Thin, faint sporulated oocyst membranes observed in this study were comparable with observations by Baneth et al. (2007).

**Maturing sporocysts and sporozoites at various stages of development** (Fig. 1e–h): Multiple sporocysts were seen, suspended free in the haemocoel, round in shape, at various stages of development. Sporocysts measured 30.34 ± 2.00 (26.48–34.19) x 29.8 ± 2.31 (24.03–32.76) *μ*m, area 714.30 ± 73.00 (532.95–825.976) *μ*m^2^ (n = 20) and contained 30 ± 6 (21–41) (n = 14) sporozoites. Distinct white membranes encapsulated immature sporocysts (Fig. 1 e). Intrasporocystic sporozoites seen at various stages of development (Fig. 1e–h). Immature sporocysts contained separating, immature sporozoites in the process of differentiating (Fig. 1 e). Maturing sporocysts containing developing sporozoites undergo membrane lysis (Fig. 1 g), membrane turquoise colour and looser around sporocyst than Fig. 1 f.

Intrasporocystic sporozoites were elongated and measured 12.86 ± 4.22 (6.56–19.92) x 2.00 ± 0.60 (1.17–2.81) *μ*m (n = 9). Nuclei measured 1.72 ± 0.56 (0.93–3.4) x 1.65 ± 0.46 (2.00–2.60) *μ*m (n = 23). Immature sporozoites differentiated at apical area of sporocysts, invaginated by thin, dark blue membranes. Immature intrasporocystic sporozoites, with uniformly blue cytoplasm (Fig. 1 e), crowned the sporocyst basal mass, light blue and foamy (Fig. 1 e yellow arrow).

Apical aggregation of dense chromatin observed within immature sporozoites, dark blue and spherical (Fig. 1 e black arrow). Immature intrasporocystic sporozoites develop to have turquoise, grainy cytoplasm (Fig. 1 f) and dense, dark blue chromatin, sometimes with purple and magenta granules (Fig. 1 f red arrow). Immature intrasporocystic sporozoite membranes in Fig. 1 f were white and more distinguishable than Fig. 1 e. Small area of light blue, foamy, basal mass was still visible in maturing sporocyst (Fig. 1 f yellow arrow). Maturing intrasporocystic sporozoites were more evenly distributed throughout sporocyst (Fig. 1 g) than Fig. 1 f, the cytoplasm of some stained grainy turquoise (Fig. 1 g). Immature intrasporocystic sporozoites were more distributed throughout the maturing sporocyst (Fig. 1 f), relaxing their previous crown formation around sporocyst basal mass (Fig. 1 e). Spongy basal mass of mature sporocyst completely absorbed, leaving only a white central area (Fig. 1 g black arrow), subsequently completely absent and replaced by mature sporozoites (Fig. 1 h).

Mature sporocyst with a lysed membrane was seen (Fig. 1 h), containing mature intrasporocystic sporozoites with distinct, white membranes and grainy light blue cytoplasm (sometimes foamy light blue (Fig. 1 h)). Mature intrasporocystic sporozoite nuclei were dense, dark blue chromatin with purple granules, rounded, and more centralized than Fig. 1f and g. Ruptured mature sporocyst with distinct, white membrane (Fig. 1 I) and exuding mature sporozoites (Fig. 1 i black arrow) observed. Mature, exuding sporozoite cytoplasms were grainy light pink and light purple, no visible membrane, and tapered towards both extremities (Fig. 1 i black arrow). Chromatin was less densely packed (Fig. 1 i) than Fig. 1e–h, purple with magenta and dark purple granules, and close to middle of mature sporozoite. Mature intrasporocystic sporozoites, still visible through sporocyst membranes (Fig. 1 i red arrow), had overall bluer hue than exuding mature sporozoites from the same sporocyst (Fig. 1 i black arrow), similar to staining properties in Fig. 1 f and h.

Free-swimming mature sporozoites were suspended in tick's haemocoel (Fig. 1i and j), with staining properties similar to exuding mature sporozoites (Fig. 1 i black arrow) (see description above). Mature sporozoites tapered towards both extremities (Fig. 1I and j), with no visible membranes or capsules. Measured 17.27 ± 0.66 (16.22–18.30) x 3.29 ± 0.53 (2.71–4.24) *μ*m, area 46.51 ± 4.36 (39.88–51.65) *μ*m^2^ (n = 7). Chromatin was loosely concentrated in rectangular shapes, in middle of mature sporozoites (Fig. 1 j red arrow), with more magenta granules than exuding mature sporozoites (Fig. 1 i black arrow).

**Remarks (sporocysts and sporozoites):** Intraoocystic sporocysts were rarely encountered during this study (Fig. 1 d), thus measurements were not taken. Vincent-Johnson et al. (1997) also reported this for *H. americanum* sporogonic stages in *A. maculatum*. Intraoocystic sporocysts of *Hepatozoon* species associated with carnivores seem to range in size from 18 to 34 μm long by 13–39 μm wide (Table 1). We did observe, however, that these intraoocystic sporocysts had a lightly blue-stained, foamy appearance and were rounded (Fig. 1 d). This shape was similar to that of *H. americanum* in *A. maculatum* (Vincent-Johnson et al., 1997; Mathew et al., 1999) and *H. canis* in *Hs. longicornis* (Murata et al., 1995), but different from the oval shaped intraoocystic sporocysts of *H. canis* in *R. sanguineus* ticks (Mathew et al., 1999; Baneth et al., 2007) and *H. ursi* in *Hs. flava* ticks (Kubo et al., 2008). Some reports on intraoocystic sporocysts from other *Hepatozoon* species indicate that these sporocysts could also be ellipsoidal (Furman, 1966; Redington and Jachowski, 1971), subspherical (Kubo et al., 2008) or round (Murata et al., 1995; Vincent-Johnson et al., 1997). Wenyon (1911) specifically reported *H. canis* intraoocystic sporocysts to also be ellipsoidal. Unlike the thick membranous walls of intraoocystic sporocysts reported for *H. americanum* in *A. maculatum* (Mathew et al., 1999), but similar to Murata et al. (1995)'s description of *H. canis* in *Hs. longicornis,* intraoocystic sporocysts from the present study had thin membranes. Morphometrically, the free sporocysts from this study were larger than that of *H. americanum* in *A. maculatum* (Vincent-Johnson et al., 1997; Mathew et al., 1998; Ewing et al., 2002), but smaller than that of *H. canis* in *R. sanguineus* (Vincent-Johnson et al., 1997; Giannelli et al., 2013b). The number of intrasporocystic sporozoites in free-lying sporocysts from the current study exceeded that of *H. americanum* in *A. maculatum* (Mathew et al., 1998; Ewing et al., 2002) and *H. canis* in *Hs. longicornis* (Murata et al., 1995) (Table 1).

Sporocyst formation appeared similar to that of other species of *Hepatozoon* (Wenyon, 1911; Allison and Desser, 1981; Van As et al., 2015). Intraoocystic sporocysts in the *Ixodes* sp. tick from the current study seemed to aggregate at the apical area of the encompassing oocyst and subsequently break out of the oocyst from there (Fig. 1 d white arrow). From a mammalian carnivore hepatozoonosis point of view, reports of mature sporocysts lying free in a tick vector's haemocoel are relatively scarce compared to reports on mature oocysts. Existing records include that of *H. canis* and *H. americanum* in an assortment of tick species (Murata et al., 1995; Vincent-Johnson et al., 1997; Mathew et al., 1998; Ewing et al., 2002; Giannelli et al., 2013a) (Table 1). McCully et al. (1975) found free lying sporocysts of an unknown *Hepatozoon* species in haemolymph smears of a *R. simus* tick collected from a spotted hyaena in the same geographical area where this study's leopard was sampled.

Free-lying sporocysts sometimes stained very dark indigo and tended to occur in thicker smear areas (Fig. 1 k), making them difficult to see on the microscope slide (Fig. 1 k white arrows). In several sporocysts, minimal intrasporocystic detail could be seen (Fig. 1 k), impeding collection of intrasporocystic, and subsequently, immature sporozoite data. Younger, immature sporocysts had more basal mass, giving it a foamy appearance (Fig. 1 d white arrow; e yellow arrow). Sporocystic basal mass (seen as degree of foaminess) seemingly decrease as intrasporocystic sporozoite counts increase (compare Fig. 1 e yellow arrow; f yellow arrow; h). Lysis of sporocyst membranes was seen as the dense, distinctly white membranes (Fig. 1e and f) seemingly loosen into faintly turquoise (Fig. 1 g) and light blue (Fig. 1 h) membranes as sporocyst matures.

Visible intrasporocystic sporozoites in free-lying sporocysts from the present study were longer, but thinner than that reported by Murata et al. (1995) for *H. canis* in *Hs. longicornis* (Table 1). Intrasporocystic sporozoites from the current study varied in size and were curved and elongated, similar to *H. canis* in *R. sanguineus* (Baneth et al., 2007) (Table 1). In the present study the staining properties of intrasporocystic sporozoites changed during maturation. The staining of intrasporocystic sporozoites lightly stained at early development, with barely visible membranes and a smooth, uniform cytoplasm with no staining granules (Fig. 1 e). Intrasporocystic sporozoites seemingly become more granulated as they mature, with more pronounced membranes (compare Fig. 1 e and h). Sporozoite formation seemed to take place through endopolygeny at the apex of each sporozoite (Fig. 1 e), with the nucleus migrating towards the middle of the intrasporocystic sporozoite as it matures (Fig. 1 f–h). Immature intrasporocystic sporozoite nuclei were rounded and similar to that described by Baneth et al. (2007) and Van As et al. (2015), but changed in shape as sporozoites matured to become more rectangular (compare Fig. 1 e black arrow; i) (Table 1). Similar to these findings, the concerning basal mass decreases as intrasporocystic sporozoites mature. Mathew et al. (1999) reported a decreased foaminess for *H. americanum* in *A. maculatum*, while the number of definably shaped sporozoites increase.

The free-lying, exuded sporozoites from the current study (Fig. 1i and j) had similar staining properties to that of *H. canis* in *R. turanicus* (Giannelli et al., 2017), with the nuclei positioned in the middle of the sporozoite body, similar to those documented for *H. americanum* by Mathew et al. (1998). However, the sporozoites from the present study were less rounded and tapered more towards its extremities, than that reported by Mathew et al. (1998) (*H. americanum*) and Giannelli et al. (2017) (*H. canis*). Free-lying sporozoites from the current study were elongated and fell morphometrically within the range of *H. americanum* in *A. maculatum* (Mathew et al., 1998), but were shorter than that of *H. canis* (Giannelli et al., 2017) (Table 1).

## 4 Discussion

Transmission of *Hepatozoon* species in leopards may occur due to ingestion of infected vectors, as is the case for *H. americanum* (Vincent-Johnson et al., 1997), *H. canis* (Baneth et al., 2007; Giannelli et al., 2013a, 2013b), *H. ursi* (Kubo et al., 2008) and possibly *H. felis* (Baneth et al., 2013). This may happen during feeding on infected prey or grooming, with infection between mother and cubs highly probable. Most ticks of southern African wildlife are multi-host species, found on both predator and potential prey species alike (Walker et al., 2003), and may act as vectors between these two parties. Leopards are known to pluck feathers and fur with their incisors from their prey before feeding (Bothma, 1998; Carnaby, 2006); also evident in the large quantity of hair usually present in leopard scat (Walker, 2007). This plucking behaviour may easily dislodge ticks from prey hosts, to be swallowed by the leopard. Due to the multiple-host species nature of wildlife associated ticks in southern Africa, another potential path of transmission may be when a leopard feeds on another infected vertebrate, ingesting tissue-associated merogonic cyst stages (Smith, 1996; Baneth et al., 2007; Baneth, 2011). Reports on *Hepatozoon* species from leopards are relatively scarce and usually in the form of molecular or microscopic detection in peripheral blood smears (Brocklesby and Vidler, 1963, 1965; Keymer, 1971; Khoshnegah et al., 2012; Pawar et al., 2012; Van As et al., 2020). McCully et al. (1975) reported on merogonic stages of an unspecified *Hepatozoon* species in an African leopard, from the same geographical region where the current study's leopard was sampled, but did not mention associated stages in ticks from that leopard. It should be noted that, during this current study, engorged ticks from several genera were collected from an infected leopard and were given the opportunity to digest blood meals before dissection.

**Fig. 2:**
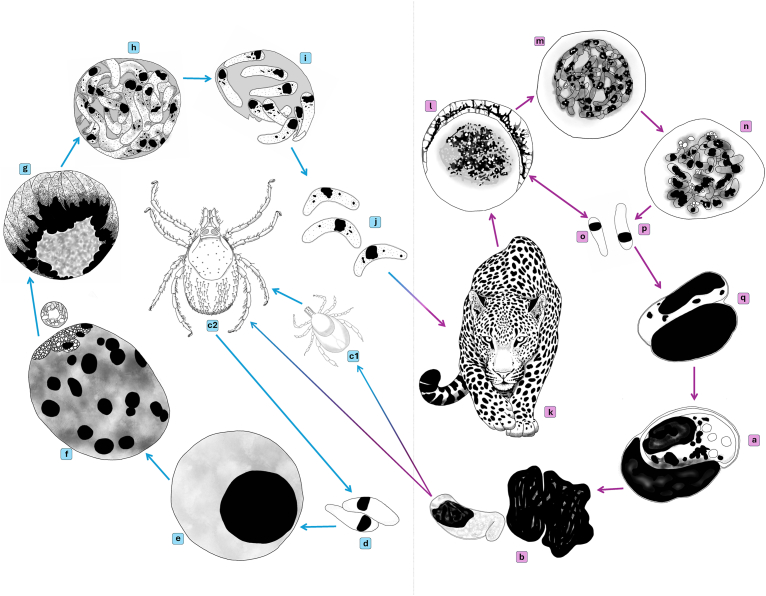
Diagram of the possible life cycle of *Hepatozoon ingwe* in its intermediate and definitive hosts. This illustration includes redrawn portrayals of merogonic stages of *Hepatozoon felis* and an unnamed species of *Hepatozoon* in spotted hyenas from the same area in South Africa as the leopard from the current study. **a and b:** Mature and extracellular gamonts in peripheral blood of the African leopard are ingested with a blood meal by an *Ixodes* species tick **(c),** either by a nymph **(c1)** (Ewing et al., 2002) (redrawn from Short and Pesapane, 2024) or by an adult **(c2); d:** Male microgamete and female macrogametes fuse to form a zygote (redrawn from Baneth et al., 2007); **e:** Zygote or early oocyst (redrawn from Baneth et al., 2007; Gianelli et al., 2017); **f:** Multi-nucleate sporulating oocyst with immature sporocysts exiting from apexal area; **g:** Young, free-lying sporocyst at the onset of sporozoite formation; **h:** Mature, free-lying sporocyst containing fully developed sporozoites; **j:** Free-lying extracellular sporozoites; **k:** African leopard ingests *Ixodes* sp. tick containing infective stages in its haemocoel; **l:** Young meront (Baneth et al., 2013); **m:** Maturing meront (Baneth et al., 2013); **n:** Mature meront (McCully et al., 1975; Baneth et al., 2013); **o:** Macromerozoite undergoes secondary merogony (O'Dwyer, 2011); **p:** Micromerozoite (O'Dwyer, 2011); **q:** Micromerozoite invades lymphocyte and grow into immature gamont. Not to scale.

Within this framework, this paper, read together with Van As et al. (2020), links both the development in the definitive and intermediate hosts of *Hepatozoon ingwe*. Usually, the identification of species of *Hepatozoon* DNA in inactive, engorged ticks, or the discovery of fully developed oocysts in the tick's haemolymph, strongly indicates the tick's role as a vector (Giannelli, 2017). The present work thus in combination with that of Van As et al. (2020) allows for the opportunity to present a detailed, illustrated representation of the possible life cycle of *Hepatozoon ingwe* (Fig. 2). Immature, mature and extracellular gamonts of *H. ingwe* from the peripheral blood smears of this leopard is shown in Fig. 2 a, b, and q. Merogonic stage sections of this illustration (Fig. 2l–p) include redrawn portrayals of merogony of *H. felis* in domestic cats (Baneth et al., 2013), and an unnamed *Hepatozoon* in spotted hyaenas (from the same area in South Africa where the leopard from the present study was sampled (McCully et al., 1975)) (Fig. 2l–p). Development via a maturing *Ixodes* tick nymph is represented by Fig. 2 b, c1 and c2 (redrawn from Short and Pesapane, 2024).

In their paper, Duplan et al. (2018) suggested that *H. silvestris* is probably transmitted by an *Ixodes ricinus* tick due to the presence of DNA in the vector. However, as pointed out by Hodžićk and Alić (2023) in the case of *H. silvestris*, the mere presence of DNA alone does not provide adequate evidence to establish the definitive role of this tick species as a biological vector. The present study has microscopically identified all corresponding sporogonic stages of a *Hepatozoon* species within a fasting Ixodes tick and molecularly confirmed that this species is indeed *H. ingwe*. This confirmation rests on the molecular characterisation of a fragment of the 18S rRNA gene, also identified in the leopard host from which the engorged tick was collected (see Van As et al., 2020). Our findings suggest that felid *Hepatozoon* are more vector specific than previously reported, given the close genetic relationship between *H. ingwe* and *H. silvestris* and the ticks belonging to the genus *Ixodes* (Van As et al., 2020; Hodžićk and Alić, 2023).

By allowing engorged ticks to be in a fasting state for seven days following collection from an infected leopard, the authors aimed to ensure that the haemogregarines already present in the potential vector's gut may undergo all possible developmental stages, if any. By doing so, the present study's method differed from that of Kubo et al. (2008) and Bhusri et al. (2017), who preserved engorged ticks in 70% ethanol immediately after collection.

*Hepatozoon ingwe* had the typical phases of sporogonic development reported for vectors that needs to be ingested by an intermediate host for the life cycle to be completed (Smith, 1996). As only adult ticks were found during this study, there would be value in future examinations of development in nymphs. A high variation with regards to the site of early sporogonic development of mammalian *Hepatozoon* species in their invertebrate hosts has been reported (Furman, 1966; Redington and Jachowski, 1971; Krampitz, 1981; Mathew et al., 1999; Baneth et al., 2007), this potentially indicating that there may be no definite migration path that is followed by all species. The authors acknowledge that tissue samples from the intermediate feline carnivore host are necessary to enable the description of a complete life cycle. Nevertheless, it is accepted that a species of tick can be regarded as a successful vector for a *Hepatozoon* species if developing and sporulated oocysts are present in its haemocoel (Ewing et al., 2002), which is what was found in this tick, together with DNA of *H. ingwe*.

It has been suggested that mammalian carnivore *Hepatozoon* species might have a narrower range of definitive (invertebrate) hosts/vectors compared to the intermediate (vertebrate) hosts (Ewing et al., 2002). However, some species, such as *H. americanum* and *H. canis,* are successfully transmitted by more than one tick species (Murata et al., 1995; O'Dwyer et al., 2001; Forlano et al., 2005; Rubini et al., 2009; de Miranda et al., 2011; Demoner et al., 2013). Only three studies report the presence of a *Hepatozoon* species, in the form of molecular detection, in engorged ticks collected from a feline carnivore. Firstly, *H. felis* was reported from *Ixodes tanuki*, collected from a Tsushima leopard cat *Prionailurus bengalensis* in Japan (Tateno et al., 2015). Secondly, Bhusri et al. (2017) reported the presence of *H. felis* in engorged *R. sanguineus* ticks infesting captive lions in Thailand. Thirdly, Duplan et al. (2018) detected *H. silvestris* in *Ixodes ricinus* in Europe. A study by McCully et al. (1975) on lions, spotted hyaenas, black-backed jackals, cheetahs and a leopard from the same geographical region as our study's sampled leopard, reported hepatozoonosis in all of these carnivores. Furthermore, McCully et al. (1975) observed sporogonic development of an unspecified *Hepatozoon* species in engorged adult *R. simus* ticks from an infected Spotted hyaena, simultaneous with no development in *R. sanguineus* or *Hs. leachi* collected from the same hyaena. Similarly, the present study also found no developmental stages in *R. sanguineus* and *Hs. leachi* ticks. Moreover, McCully et al. (1975) did find that *R. sanguineus* are successful vectors for a *Hepatozoon* sp. infecting Black-backed jackals. The above reports may suggest that arthropod vectors may be more species-specific to transmit species of *Hepatozoon* than previously assumed, especially in the wild. Since sporogony is evident from the current study's results, it can be assumed that oogenesis occurred in this specific *Ixodes* tick (Siddall, 1995; Smith, 1996), but not in the other screened ticks. The absence of gametogenesis, syzygy and subsequent fertilization, may indicate these stages to have been completed by the time the tick was dissected, as these stages may be observed as early as 24 h after a blood meal (Baneth et al., 2007). This, together with reports of other leopards infected with *H. ingwe* (Van As et al., 2020), may indicate that, at least for this specific species of *Hepatozoon*, naturally occurring *Ixodes* ticks may be successful vectors between different leopards in the same areas.

Oocyst and sporocyst shape seems to show intraspecific variation, dependent on the tick vector species. This can be seen in *H. canis*, which has round oocysts in *A. ovale* (Forlano et al., 2005; Rubini et al., 2009) and oval oocysts in *R. microplus* (de Miranda et al., 2011), *R. sanguineus* (Giannelli et al., 2013b) and *R. turanicus* (Giannelli et al., 2017). *Hepatozoon canis* sporocysts could be elongated, oval or ellipsoidal in different tick vectors (Forlano et al., 2005; Baneth et al., 2007; Giannelli et al., 2013a, 2013b). Round sporocysts, similar to those observed in the present study (Fig. 1e–i), were observed and reported in *R. simus* (unknown *Hepatozoon* species) (McCully et al., 1975).

Most mammalian carnivore life cycle studies suggest that oocysts in the haematophagous vector are the infective stage to the vertebrate host that ingests that vector (Baneth et al., 2001, 2007; Baneth, 2011), and rarely go beyond morphological description of oocysts. The current study adds value by morphologically describing sporocysts and sporozoites and various stages of development. Previous studies have shown that contact with bile induces oocysts to rupture, which led several authors to conclude that mature sporulated oocysts are the infective stage of carnivore *Hepatozoon* to their intermediate hosts (Redington and Jachowski, 1971; Mathew et al., 1999). Thin, faint sporulated oocyst membranes observed in the current study may indicate a readiness to tear easily so that sporocysts may be released (Baneth et al., 2007). However, the presence of free mature sporocysts and free sporozoites found in the present study may indicate that more immediate infective stages may also be ingested with the haematophagous vector. We propose that this may lead to faster, more successful infection in the intermediate carnivore host.

No intrasporocystic sporozoites were seen in intraoocystic sporocysts during the present study, but details from other carnivore-associated *Hepatozoon* species can be seen in Table 1. Other studies did report on their morphological characteristics and it seems that this developmental stage in carnivore-associated *Hepatozoon* species measures 10–15 μm long by 3–5 μm wide (Mathew et al., 1999; Kubo et al., 2008). According to available reports, intraoocystic sporocysts of carnivore-associated *Hepatozoon* may contain 8–26 sporozoites each (Table 1).

Sporocysts from the present study seemed to follow a path of maturation in line with descriptions by Mathew et al. (1999) and Baneth et al. (2007), and the author proposes that the degree of maturation can be classified from immature to mature according to the following characteristics: As immature sporocysts develop further, the amount of sporocystic basal material (seen as foaminess) decrease while the number of immature, differentiating intrasporocystic sporozoites increase. As this process continues, lysis of the sporocyst membrane increases, seen in changing staining properties and membranes seemingly loosening and becoming fainter. Thus, immature sporocysts will have an overall foamy appearance with a thick membrane, while mature sporocysts will have sporozoites that are distributed throughout the whole cytoplasm, with no basal material and a lysed, faint membrane. The current study proposes that these characteristics could dependably be used to distinguish between different sporocysts at different stages of maturation, within one vector.

The present study is the first to report intrasporocystic sporozoite nuclei morphometrics of a carnivore-associated *Hepatozoon*, as well as free-lying sporozoites in the haemocoel of a naturally infected tick collected from a naturally infected carnivore host. Sporozoite development followed the same path/trend/pattern as that of *H. americanum* (Mathew et al., 1999), with nuclei changing from rounded, densely chromatinised areas in immature sporozoites to rectangular, loosely chromatinised areas with purple and magenta granules in mature, exuded sporozoites. Our study suggests these characteristics to be a good diagnostic means of determining the degree of development of sporozoites within a tick vector.

Reports on life cycle stages from wild ticks collected off wild animals are quite rare (Ewing et al., 2002; de Miranda et al., 2011), which is where the present study adds value to existing knowledge. The definitive answer as to which ticks may act as vectors for feline hepatozoonosis remains relatively unanswered for big cats in southern Africa, and this study provides some valuable morphological data in this regard, showing the importance thereof when coupled with genetic confirmation of haemogregarines in haematophagous vectors. In the case of this study, and due to the current conservation status of the African leopard in South Africa, we were not permitted to kill and dissect a specimen and during the study no specimens became available that either died from natural causes or as a result of human-wildlife conflict. Subsequently, a detailed description of the merogonic stages of *Hepatozoon ingwe* is still lacking. The current study sheds light on a possible tick vector in which almost all developmental and infective stages of *Hepatozoon ingwe* was observed, but further in-depth studies are necessary to determine the complete life cycle of this haemogregarine. Understanding the transmission process of hepatozoonosis in large carnivores provides a good basis for further research into the ecological and health impacts of this infection, ultimately aiding in the conservation management of large carnivores.

## CRediT authorship contribution statement

**Michelle van As:** Writing – review & editing, Writing – original draft, Visualization, Project administration, Methodology, Investigation, Formal analysis, Data curation, Conceptualization. **Edward C. Netherlands:** Writing – review & editing, Resources, Methodology, Investigation, Formal analysis, Data curation. **Johann van As:** Writing – review & editing, Visualization, Validation, Methodology, Investigation, Formal analysis, Conceptualization. **Courtney A. Cook:** Writing – review & editing, Writing – original draft, Supervision, Investigation, Conceptualization. **Nico J. Smit:** Writing – review & editing, Writing – original draft, Validation, Supervision, Resources, Project administration, Investigation, Funding acquisition, Conceptualization.

## Declaration of competing interest

The authors declare that they have no known competing financial interests or personal relationships that could have appeared to influence the work reported in this paper.
